# Recombinant human soluble thrombomodulin improves mortality in patients with sepsis especially for severe coagulopathy: a retrospective study

**DOI:** 10.1186/s12959-018-0172-6

**Published:** 2018-08-24

**Authors:** Takahiro Kato, Katsuhiko Matsuura

**Affiliations:** 1 Departments of Pharmacy, Aichi Medical University, 1 -1 Yazakokarimata, Nagakute, Aichi 480-1195 Japan; 2 Laboratory of Clinical Pharmacodynamics, Aichi Gakuin University School of Pharmacy, Nagakute, Japan

**Keywords:** Thrombomodulin, Sepsis, Disseminated intravascular coagulation, Severe coagulopathy

## Abstract

**Background:**

Disseminated intravascular coagulation (DIC) is associated with high mortality in patients with sepsis. Several studies reporting that recombinant human soluble thrombomodulin (rhTM) reduced mortality in sepsis patients. This retrospective cohort study aimed to evaluate the efficacy of rhTM for patients with mild coagulopathy compared with those with severe coagulopathy.

**Methods:**

We evaluated about 90-day mortality and SOFA score. SOFA score was also evaluated for the following components: respiratory, cardiovascular, hepatic, renal and coagulation.

**Results:**

All 69 patients were diagnosed with sepsis, fulfilled Japanese Association for Acute Medicine criteria for DIC, and were treated with rhTM. Patients were assigned to either the mild coagulopathy group (did not fulfill the International Society on Thrombosis and Haemostasis overt DIC criteria) or the severe coagulopathy group (fulfilled overt DIC criteria). The 90-day mortality was significant lower in severe coagulopathy group than mild coagulopathy group (*P* = 0.029). Although the SOFA scores did not decrease in the mild coagulopathy group, SOFA scores decreased significantly in the severe coagulopathy group. Furthermore the respiratory component of the SOFA score significant decreased in severe coagulopathy group compared with mild coagulopathy group.

**Conclusions:**

rhTM administration may reduce mortality by improving organ dysfunction especially for respiratory in septic patients with severe coagulopathy.

## Background

Sepsis and septic shock remain the most common cause of death in critically ill patients [1], and new therapeutic approaches are urgently needed. Ideal management of sepsis and septic shock remains controversial. Current evidence suggests that compliance with the Surviving Sepsis Campaign (SSC) guidelines is associated with decreased mortality [2–4]. Furthermore, it has been reported recently that early lactate clearance has a lower mortality risk than early goal-directed therapy [5].

Disseminated intravascular coagulation (DIC) is associated with high mortality in patients with sepsis [6]. Excessive coagulation activation, inhibition of fibrinolysis, and consumption of coagulation inhibitors lead to a hypercoagulable state, resulting in fibrin deposition in microvessels and inflammatory reactions [7]. Current management of DIC is primarily focused on treating any associated underlying medical condition, although use of supplemental clotting factors or platelets, or anticoagulant therapy may occasionally be required [8]. In particular, therapeutic intervention directly against coagulation and inflammation for DIC associated with sepsis is effective [9, 10], and it is generally accepted that early, aggressive treatment of the underlying disease is important. The revised Japanese Association for Acute Medicine (JAAM) criteria allows diagnosis of DIC in the earlier phase of disease than the overt DIC criteria published by the International Society on Thrombosis and Haemostasis (ISTH) [11].

Recombinant human soluble thrombomodulin (rhTM) is the only agent for the treatment of DIC [12]. rhTM was approved in 2008 and has been used clinically for DIC treatment in Japan. Several animal studies have demonstrated a reduction in mortality with the administration of rhTM in sepsis models [13]. Moreover, rhTM prevents endotoxin-induced lung injury in rats by leukocyte activation [14]. Multicenter retrospective study and meta-analysis have shown that rhTM improves mortality in sepsis patients [15, 16]. Yamakawa et al. shown that rhTM improves coagulopathy and decreases SOFA score compared with control group in patients with sepsis [17].

We hypothesized that mortality would be improved after treatment with rhTM in septic patients especially for mild coagulopathy. Therefore, the purpose of this study was to evaluate the efficacy of rhTM for patients with mild coagulopathy compared with those with severe coagulopathy.

## Methods

### Patients and study design

**Fig. 1:**
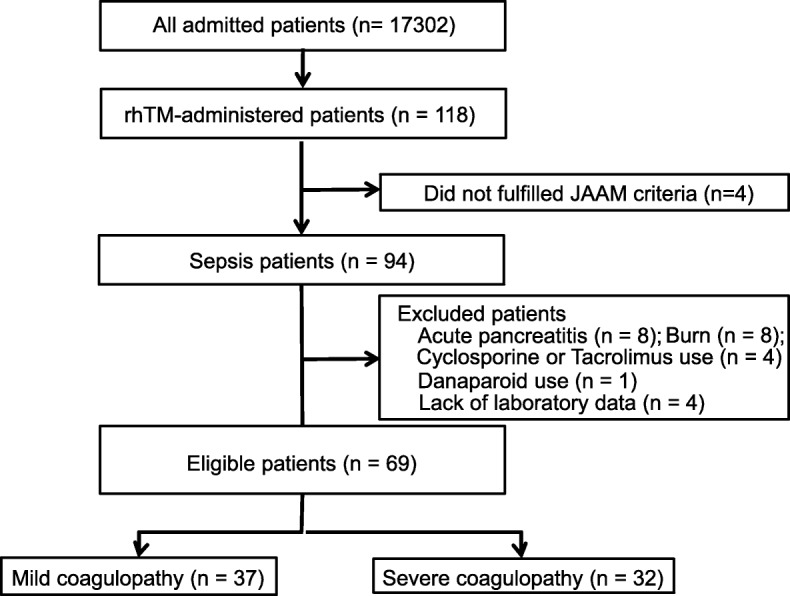
Flow diagram of patient inclusion and exclusion criteria. rhTM, recombinant human soluble thrombomodulin. JAAM, Japanese Association for Acute Medicine

This was a retrospective cohort study. All patients were admitted to Aichi Medical University Hospital intensive care unit (ICU) between May 2008 and and December 2014. Although the criteria for ICU admission were not standardized, all patients included in this study were diagnosed with sepsis what is according to SEPSIS-3 [18] and DIC diagnosed by JAAM criteria (Score ≥ 4) [11] (Table 1), and were treated with rhTM. rhTM doses were 0.06 mg/kg/day (or 0.02 mg/kg/day for patients who required renal replacement therapy for acute kidney injury [AKI]); rhTM was administered for 30 min once daily. All patients were principally treated according to the SSC guidelines [19]. The exclusion criteria were: acute pancreatitis, burns, treatment with danaparoid sodium at the start of treatment, treatment with cyclosporine or tacrolimus until sepsis diagnosis, age ≤ 15 years, and those not diagnosed with DIC due to a lack of laboratory data. A flow diagram of patient inclusion is shown in Fig. 1. The study protocol was reviewed and approved by an institutional review board. Informed consent was not required because blood samples were taken as part of the routine patient care for clinical laboratory testing, but the highest standard of privacy policy was applied.

**Table 1:** JAAM criteria [11] and ISTH overt DIC criteria [22]

	JAAM		ISTH		
Score	1	3	1	2	3
SIRS	≥3 items				
Platelet Count (10^3^/mL)	≥80 and < 120, or > 30% decrease within 24 h	< 80 or 50% decrease within 24 h	< 100	< 50	
FDP (μg/mL)	≥10 and < 25	≥25		≥10 and < 25 (Moderate increase)	≥25 (Strong increase)
PT Ratio	≥1.2				
Prolonged prothrombin time (sec)			3<, < 6	6<	
Fibrinogen level (g/L)			< 1.0		
Diagnosis	4 points or more		5 points or more		

These score was assessed using d-dimer if FDP was not measured. The cut off value of d-dimer level were “moderate increase; 5.4–13.2 μg/mL, strong increase; ≥13.2 μg/mL”

JAAM: The revised Japanese Association for Acute Medicine

ISTH: The International Society on Thrombosis and Haemostasis

SIRS: systemic inflammatory response syndrome

Criteria for SIRS (systemic inflammatory response syndrome)

• Temperature: > 38 °C or < 36 °C

• Heart rate: > 90 beats/min

• Respiratory rate: > 20 breath /min or PaCO2 < 32 Torr (< 4.3 kPa)

• White cell blood counts: > 12,000/mm3, < 4,000cells/mm3, or 10% immature (band) forms

### Data collection

Relevant clinical background, medication history, and laboratory data of all patients were collected at appropriate times during the treatment for sepsis. We collected the patients’ demographic and laboratory test data, including age, sex, clinical outcome (mortality at 90 days), shock (hypotension not reversed with fluid resuscitation), presence of AKI as defined by the AKI network [20], acute physiologic and chronic health evaluation II (APACHE II) score, sequential organ failure assessment (SOFA) score (Table 2) [21], presence of DIC as defined by the ISTH overt DIC criteria [22], mechanical ventilation, renal replacement therapy, vasopressor use, presence of cancer, prothrombin time (PT) ratio, antithrombin III (AT III) activity, D-dimer level, platelet count, fibrinogen level and lactate level. The SOFA score was recorded on days 1, 3, 5, 7 and 28 after administration of rhTM.

**Table 2:** SOFA score [21]

SOFA score	1	2	3	4
Respiratory PaO2/FiO2, mmHg	< 400	< 300	< 200 (With respiratory support)	< 100 (With respiratory support)
Coagulation Platelets × 103/μl	< 150	< 100	< 50	< 20
Hepatic Bilirubin, mg/dl	1.2–1.9	2.0–5.9	6.0–11.9	> 12.0
Cardiovascular (Adrenergic agents dose is in μg/kg/min)	MAP < 70 mmHg	Dopamine ≤5 or dobutamine (any dose)	Dopamine > 5 or epinephrine ≤0.1 or norepinephrine ≤0.1	Dopamine > 15 or epinephrine > 0.1 or norepinephrine > 0.1
Central nervous system	13–14	10–12	5–9	< 6
Renal Creatinine, mg/dl or Urine output	1.2–1.9	2.0–3.4	3.5–4.9 Or < 500 ml/day	> 5.0 Or 200 ml/day

### Evaluation of clinical response

Patients were assigned to either the severe coagulopathy group or the mild coagulopathy group. Severe coagulopathy was defined as diagnosed DIC by not only JAAM criteria (Score ≥ 4) but also ISTH overt DIC criteria (Score ≥ 5). Mild coagulopathy was defined as fulfilled the JAAM criteria only (Table 1).

We evaluated about 90-day mortality and SOFA score at day 1, 3, 5, 7 and 28. An animal study has been reported that rhTM prevents endotoxin-induced lung injury [14]. Therefore SOFA score was also evaluated for the following components: respiratory, cardiovascular, hepatic, renal and coagulation. Moreover, changes over time in coagulation test results (D-dimer level, PT ratio and fibrinogen level) were assessed from day 1 to day 7. PT and fibrinogen were measured by electric impedance methods (PT: Thrombocheck PT; Sysmex CO., LTD., fibrinogen: Coagpia®Fbg; Sekisui medical CO., LTD.). FDP and D-dimer were measured by latex nephelometric immunoassay (Nanopia®P-FDP; Sekisui medical CO., LTD., LIAS AUTO® D-Dimer NEO; Sysmex CO., LTD.). AT III activity was measured by synthetic substrate method (Testzym®S AT III; Sekisui medical CO., LTD.).

### Statistical analysis

Data were expressed as group mean ± standard deviation, or percentages. Continuous variables were compared between groups using the Student’s *t*-test. Noncontinuous variables were compared between groups using the Mann–Whitney U test. Categorical variables were analyzed using the chi-squared test or Fisher’s exact test. Log-rank analysis was used to evaluate 90-day mortality. Multivariate Cox regression analysis was used to assess the covariates that were associated with time to mortality. SOFA scores, Platelet count, D-dimer level, PT ratio and fibrinogen level between groups over time were analyzed by repeated measures analysis of variance and post hoc Dunnett’s test. Independent predictive variables with a *P* value of less than 0.05 were considered statistically significant. Statistical analyses were performed using JMP for Windows version 5.0.1 software (SAS Institute, Inc., U.S.).

## Results

### Baseline characteristics

Although 94 patients were diagnosed with sepsis and treated with rhTM during the study period, only 69 patients met the requirements of our study (Fig. 1). Thirty-seven patients were in the mild coagulopathy group and 32 patients were in the severe coagulopathy group. The characteristics of the study population are shown in Table 3. FDP levels were not measured in most of patients. ISTH overt DIC score, JAAM DIC score, D-dimer level and Platelet count were significant difference between mild coagulopathy group and severe coagulopathy group. These score was assessed using d-dimer if FDP was not measured. The cut off value of d-dimer level were “moderate increase; 5.4-13.2 μg/mL, strong increase; ≥13.2 μg/mL”.

**Table 3:** Characteristics of patients

	Mild coagulopathy (*n* = 37)	Severe coagulopathy (*n* = 32)	*P* value
Male, n (%)	25 (67.6)	23 (71.9)	0.796
Age (years)	69 [38–94]	72.5 [33–91]	0.110
Shock, n (%)	14 (42.4)	13 (40.6)	1.000
AKI, n (%)	15 (42.9)	18 (56.3)	0.332
APACHE II score	26.6 ± 8.1	29.8 ± 10.8	0.229
SOFA score	6.2 ± 3.5	7.5 ± 2.5	0.114
ISTH overt DIC score	3.4 ± 0.7	5.3 ± 0.6	< 0.001
Mechanical ventilation, n (%)	31 (88.6)	30 (93.8)	0.675
Renal replacement therapy, n (%)	25 (71.4)	22 (68.8)	1.000
Vasopressor use, n (%)	24 (68.6)	24 (75.0)	0.598
Lactate (mmol/L)	32.0 ± 22.8	38.3 ± 21.5	0.289
Time for normalize lactate level (h)	94.8 ± 142.6	88.3 ± 87.0	0.854
Cancer, n (%)	6 (16.7)	5 (15.6)	1.000
rhTM dose (mg/kg)	0.041 ± 0.018	0.048 ± 0.023	0.218
Duration of rhTM administration (days)	5.4 ± 1.8	6.3 ± 3.5	0.181
Coagulation tests			
Prothrombin time ratio	1.34 ± 0.28	1.42 ± 0.26	0.212
Antithrombin III activity (%)	57.7 ± 20.6	54.5 ± 21.8	0.581
D-dimer (10^3^ ng/ml)	14.0 ± 15.4	40.0 ± 65.0	< 0.001
Platelet count (10^3^/μl)	10.0 ± 10.1	5.3 ± 3.2	0.004
Fibrinogen (mg/dl)	420.3 ± 216.7	410.0 ± 117.7	0.813

Collected data are when rhTM administration start

Data are presented as mean ± standard deviation unless otherwise stated. DIC: disseminated intravascular coagulation, *AKI*: acute kidney injury, *APACHE II:* acute physiologic and chronic health evaluation, *SOFA*: sequential organ failure assessment, *ISTH*: International Society on Thrombosis and Haemostasis, rhTM: recombinant human soluble thrombomodulin

### Influence of treatment for mild or severe coagulopathy on mortality

**Fig. 2:**
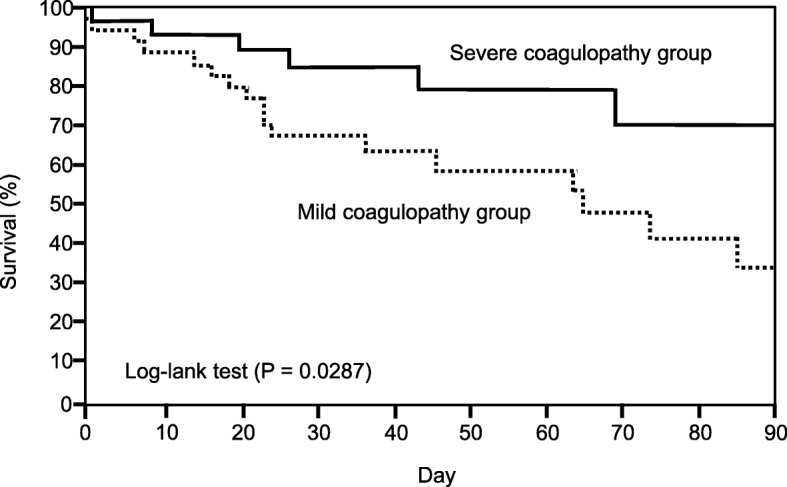
Kaplan–Meier plot of survival at 90 days The solid line represents patients in the severe coagulopathy group, and the dotted line represents patients in the mild coagulopathy group. The mortality rate was significantly different between the two groups.

The Kaplan–Meier plot of survival function during the 90-day study period is given for both the mild coagulopathy group and the severe coagulopathy group in Fig. 2. There was trend toward lower 90-day mortality in the severe coagulopathy group than mild coagulopathy group (*P* = 0.029). We performed a Cox regression analysis to assessed four possible confounders related to outcome: age, APACHE II score, SOFA score (day1) and severe coagulopathy. Consequently, severe coagulopathy influenced lower mortality (*p* = 0.015). (Table 4).

**Table 4:** Cox regression analysis

	Risk ratio	95% CI	*P* value
Age	0.985	0.950–1.024	0.422
APACHE II score	0.996	0.934–1.060	0.909
SOFA score (day 1)	1.171	0.985–1.411	0.074
Severe coagulopathy	0.554	0.314–0.897	0.015

*CI*: confidence intervals*, SOFA*: sequential organ failure assessment, *APACHE*: acute physiologic and chronic health evaluation

### Sequential organ failure assessment score

**Fig. 3:**
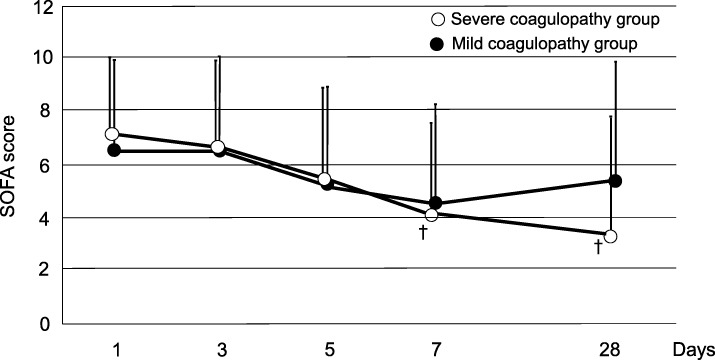
Serial changes in the sequential organ failure assessment (SOFA) score in the severe coagulopathy group and in the mild coagulopathy group Open circle: Severe coagulopathy group. Filled circle: Mild coagulopathy group. Data are expressed as means ± standard deviation. ^†^Significant difference compared with day 1**.** There was trend toward lower SOFA scores in severe coagulopathy group than mild coagulopathy group on day 28 (*p* = 0.08).

Although the SOFA score did not decrease in the mild coagulopathy group, it decreased significantly in the severe coagulopathy group at day7 and 28 (*P* = 0.001). There was trend toward lower SOFA scores in severe coagulopathy group than mild coagulopathy group on day 28 (*p* = 0.08) (Fig. 3). The respiratory component of the SOFA score significant decreased in severe coagulopathy group (*p* = 0.029) (Table 5).

**Table 5:** The components of the SOFA score at day 7

	Mild coagulopathy (*n* = 37)	Severe coagulopathy (*n* = 32)	*P* value
Respiratory	0.93 ± 0.18	0.44 ± 0.18	0.029
Cardiovascular	0.83 ± 0.25	1.08 ± 0.27	0.438
Hepatic	1.50 ± 0.29	1.43 ± 0.30	0.843
Renal	1.12 ± 0.25	0.78 ± 0.26	0.673
Coagulation	1.54 ± 0.19	1.23 ± 0.20	0.253

Data are presented as mean ± standard error unless otherwise stated

### Coagulation tests

Prothrombin time ratio and D-dimer were improved in the severe coagulopathy group significantly compared with baseline. Although D-dimer level was higher in the severe coagulopathy group than mild coagulopathy group at day 1, D-dimer was not different between two groups.

**Fig. 4:**
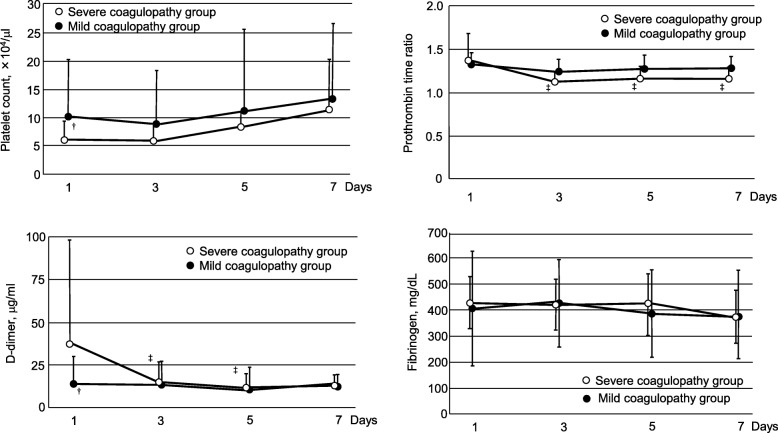
Serial changes in coagulation tests in the early phase of DIC group and in the overt DIC group Open circle: Severe coagulopathy group. Filled circle: Mild coagulopathy group. Data are expressed as means ± standard deviation. ^†^Significant difference compared with the severe coagulopathy group. ^‡^Significant difference compared with day 1.

Platelet count improved in both groups (not significant). Platelet count, Prothrombin time ratio, D-dimer and fibrinogen levels were not significantly different at day 7 between the groups. (Fig. 4).

## Discussion

Although several trials have shown that rhTM reduced mortality in septic patients with DIC [13, 14, 17], the timing of treatment initiation was unclear. The present study represents the first attempt to evaluate efficacy of rhTM in septic patients with different degree of coagulopathy. This study suggested that treatment with rhTM in septic patients with severe coagulopathy improves mortality compared with those without severe coagulopathy.

The present study included 69 patients with sepsis. There was no difference in baseline characteristics except coagulation tests between severe coagulopathy group and mild coagulopathy group. Platelet count was significantly lower and D-dimer level was significantly higher in the severe coagulopathy group than mild coagulopathy group. The sensitivity of a low fibrinogen level for the diagnosis of DIC according to ISTH criteria was 28% and hypofibrinogenemia has been detected in only severe cases of DIC [8]. Fibrinogen levels were normal in most of patients in the present study. Therefore, fibrinogen levels showed no significant differences between the two groups. PT ratio was improved significantly in the severe coagulopathy group at day 7. There was significant reduction of SOFA score in severe coagulopathy group within 7 days compared with baseline. Furthermore, at day 7 and 28, the score was trends in lower of the severe coagulopathy group, as compared with mild coagulopathy group. On the other hand, there was no significant reduction of SOFA score in the mild coagulopathy group compared with baseline.

Severe coagulopathy is associated with high mortality in patients with sepsis. Patients with severe coagulopathy had higher mortality than those with mild coagulopathy [6, 23]. The phase II study shown that rhTM trend toward to improve mortality compared with placebo in patients with sepsis [24]. The majority of patients included in the study were not severe coagulopathy state (ISTH DIC score < 5). It was considered in the study that rhTM may be more beneficial for subjects with greater coagulation abnormality. Multicenter retrospective study has shown that rhTM improved mortality significantly [16]. Yoshimura et al. reported that rhTM improves mortality in high risk septic patients (APACHEII 24–29). On the other hand, rhTM did not improve mortality in moderate risk of septic patients (APACHE II score < 24) [25]. Moreover ISTH score was significantly higher in high risk and very high risk of septic patients compared with moderate risk of septic patients. In addition, the patients included in this study were high risk of septic patients (APACHE II score; 27.1 ± 8.1 and 29.4 ± 11.0).

Generally, it is difficult to determine the survival benefit of a particular lifesaving therapy in a set of patients with a low risk of mortality. This may be one of the reasons why thTM administration does not reduce the mortality risk in patients who are not at high risk in the first place. On the other hand, these results are congruent with recent pathophysiological findings concerning the innate immune response. Under certain circumstances, thrombosis is considered to play a major physiological role, which is specifically named immunothrombosis, in immune defense [26]. However, aberrant of uncontrolled activation of immunothrombosis is likely to constitute a key event in the development of thrombotic disorders [27]. In patients in the mild coagulopathy state, rhTM could have inhibited host-defensive thrombosis, which suppress to capture and ensnare pathogens circulating in the blood, and therefore failed to improve mortality. In contrast, immunothrombosis could have been aberrantly activated and proved detrimental to the host in patients in the severe coagulopathy state, which may have improved mortality. SOFA score was trends in lower of the severe coagulopathy group, as compared with mild coagulopathy group. It is considered that rhTM may improve organ dysfunction by improving severe coagulopathy. Furthermore, respiratory component of the SOFA score was significant reduction in the severe coagulopathy group than mild coagulopathy group in the present study.

Several studies have shown that rhTM improves coagulopathy in disseminated intravascular coagulation [28, 29].

Yamakawa et al. showed that rhTM improves coagulopathy, mortality and SOFA score compared with control group [17]. It was discussed in the study that suppressing the hypercoagulative state by rhTM administration may potentially prevent the progression to multiple organ failure. rhTM has the effect of directly combining with thrombin. Thrombin – rhTM complex activates protein C. Therefore the anticoagulative effect of rhTM depends on the amount of thrombin available. The subgroup analysis of PROWESS trial has shown that activated protein C improves respiratory dysfunction in patients with sepsis [30]. Moreover, Ogawa et al., reported the respiratory component of the SOFA score reduced significantly in patients with DIC who treated by rhTM compared with placebo [31]. The results of this study indicate that respiratory component of the SOFA score was more reduced in the severe coagulopathy group than the mild coagulopathy group. rhTM was considered to improve respiratory dysfunction by more activating protein C in severe coagulopathy state than mild coagulopathy state. Therefore, administrating rhTM might more improve not only coagulopathy but also respiratory dysfunction in patients with severe coagulopathy compared with those without rhTM. We considered that there might be relation among SOFA score and mortality, SOFA score was not selected as covariate for mortality in current study. We considered reason of above was because of small sample size.

We acknowledge several limitations of our observational study design. First, this study was retrospective small scale trial. Second, this study is not a randomized placebo controlled trial. Multiple unmeasured variables might account for the outcome differences observed in this study. Third, this study was carried out in a single institution. Further multicenter, prospective randomized trials are needed to thoroughly evaluate the effects of rhTM on the treatment of septic patients with coagulopathy.

## Conclusion

In conclusion, we found that rhTM administration may reduce mortality by improving organ dysfunction especially for respiratory in septic patients with severe coagulopathy. The present study represents the first attempt to evaluate efficacy of rhTM in septic patients with different degree of coagulopathy. Further clinical investigations are necessary to evaluate the effect of rhTM in several degree of coagulopathy.

## Abbreviations

AKI: Acute kidney injury
APACHE II: Acute Physiologic and Chronic Health Evaluation II
AT III: Antithrombin III
DIC: Disseminated intravascular coagulation
ICU: Intensive care unit
ISTH: Subcommittee of the International Society on Thrombosis and Haemostasis
JAAM: Japanese Association for Acute Medicine
PT: Prothrombin time
rhTM: Recombinant human soluble thrombomodulin
SOFA: Sequential Organ Failure Assessment
SSC: Surviving sepsis campaign
